# Outcomes after Radical Prostatectomy in Ghanaians: A Surgeon's Early Experience

**DOI:** 10.1155/2013/832496

**Published:** 2013-04-24

**Authors:** Mathew Yamoah Kyei, Edward James Mensah, Samuel Gepi-Attee, Devine Kwami, Kwabena Ampadu, Emmanuel Asante, George Oko Klufio, Edward Donkoh Yeboah

**Affiliations:** ^1^Department of Surgery and Urology, University of Ghana Medical School, P.O. Box 4236, Accra, Ghana; ^2^Department of Anaesthesia, Korle Bu Teaching Hospital, P.O. Box 77 Korle Bu, Accra, Ghana

## Abstract

*Background*. There is a lack of expertise in the procedure of open radical retropubic prostatectomy in West Africa therefore necessitating the training of urologists in the subregion in this procedure. *Aim*. This report looks at the early outcomes of a single surgeon in this procedure after an SIU fellowship. *Methodology*. A prospective study of the initial twenty consecutive patients with clinically localized prostate cancer that underwent open radical retropubic prostatectomy at the Korle Bu Teaching hospital, Accra. *Results*. The mean followup was 19.5 months (range 7 months–36 months). The mean age was 62.7 yrs. For the clinical stage, 60% were T1c and 40% T2a with a mean Gleason score of 6.5. The mean estimated blood loss was 1140.0 mLs with a transfusion rate of 70%. For the pathologic stage, pT2 cancers formed 60%, pT3 25%, and pT4 5% with a mean Gleason score of 6.8. No lymph node involvement was noted. The perioperative complications rate was 15%, a postoperative potency recovery rate of 78.6% with all the patients being continent of urine. The tPSA of 95% of the patients had remained less than 0.4 ng/mL. *Conclusion*. The SIU scholarship offers an avenue for training in radical prostatectomy for sub-Saharan Africa.

## 1. Introduction

The risk factors for the development of prostate cancer include men of African ancestry [1] with high incidence rate reported in some sub-Saharan countries including Ghana, 7% in men between 50 yrs and 74 yrs [2] and Nigeria with incidence rate of 127 per 100,000 population [3]. In Ghana, prostate cancer has been found to be the second commonest cause of cancer death in men [4]. With the wide-spread use of serum prostate specific antigen (PSA) screening for prostate cancer in urban areas, it is expected that more cases of early prostate cancer will be diagnosed.

Radical prostatectomy has remained the gold standard for treatment of organ confined prostate cancer [5]. Though radical prostatectomy could be cost effective, it has been noted that access of patients with localized prostate cancer to radical prostatectomy in the management of prostate cancer in sub-Saharan Africa is low mainly due to lack of expertise in the procedure of open radical prostatectomy [6].

 In Ghana, localized prostate cancer had been managed with external beam radiotherapy and recently brachytherapy. The role of radical prostatectomy has been limited even though majority of the patients present with lower urinary tract obstructive symptoms or acute retention of urine. This is mainly due to lack of expertise. 

In 2006, a program to train urologists in Senegal in transperineal prostatectomy was commenced [6]. However, its impact on the rest of sub-Saharan Africa including Ghana has been limited. Other ways of training urologists in the West African subregion in the procedure of radical prostatectomy have become apparent. The Societe Internationale d'Urologie (SIU) fellowship is an alternate means of getting urologist in the subregion to get the needed training in other centers on the continent where radical prostatectomy for prostate cancer is routinely performed. This report looks at the early oncological and functional outcomes and morbidity of a single surgeon in the procedure of radical retropubic prostatectomy at the Korle Bu Teaching Hospital Accra after a six month SIU sponsored fellowship at University of Stellenbosch, Tygerberg Hospital, South Africa.

*Objective*.  To ascertain the patient characteristics, tumour characteristics, and the early outcomes of the initial twenty consecutive patients of an SIU scholar after training in the procedure of anatomic radical retropubic prostatectomy for localized prostate cancer. 

## 2. Materials and Methods

A prospective study of consecutive patients with clinically localized prostate cancer (cT1-cT2) that underwent open anatomic radical retropubic prostatectomy from January 2010 to July 2012 at the Korle Bu Teaching Hospital, Accra.

Patients who had a systematic transrectal ultrasound guided biopsy (12 core biopsies) which confirmed prostate cancer were staged clinically. The staging investigations included radioactive bone scan to rule out bony metastasis and a pelvic CT scan to rule out extracapsular extension of the prostate cancer. Patients diagnosed with localized prostate cancer and consented to undergo open radical retropubic prostatectomy were included in the study.

The preparation for the procedure involved admitting the patients two days before the procedure, light diet on preoperative day 2, and fluid only diet on preoperative day 1. They had phosphate enema at 6 pm on preoperative day 1 and a rectal wash out on the morning of the operation. 

Two units of homologous blood were cross-matched and made available in the operative theatre.

The patients underwent an open anatomic radical retropubic prostatectomy with obturator lymphadenectomy using a lower midline extra peritoneal incision under general anaesthesia and an epidural placed for postoperative analgesia. Antibiotic usage comprised 500 mg levofloxacin at induction and continued for 72 hours after the procedure. The prophylaxis against deep vein thrombosis involved the use of Ted stockings and subcutaneous clexane 40 mg daily for 5 days.

The parameters recorded included the patient age, clinical presentation, preoperative total prostate specific antigen (tPSA), the clinical stage, and Gleason score. Also recorded were the operative time, the estimated blood loss, the prostate size, and any blood transfusions administered. The pathological stage of the prostate cancer and perioperative complications were also noted. Patients were discharged on postoperative day 8, and the urethral catheters removed between postoperative day 14 and 21.

The followup involved three monthly clinical examinations and serial PSA determinations with tPSA greater than 0.4 ng/mL being viewed as biochemical progression.

The data was analyzed using the Statistical Package for the Social Sciences (SPSS) for Windows (Version 19.0).

## 3. Results

The initial twenty consecutive cases of open radical retropubic prostatectomy performed were considered for this report. The mean followup was 19.5 months (range 7 months–36 months).

The mean age of the patients was 62.7 yrs ± 5.6 (range 51 yrs–72 yrs). The commonest presentation was lower urinary tract obstructive symptoms (40%) (Table 1).

The mean preoperative tPSA was 16.12 ng/mL ± 13.68 ng/mL (range 2.45–62.20 ng/mL) with 1 (5%) with tPSA less than 4.0 ng/mL, 7 (35%) tPSA 4.1–10.0 ng/mL, 8 (40%) with tPSA 10.1–20.0 ng/mL, and 4 (20%) with tPSA >20.1 ng/mL. 

The mean Gleason score was 6.5 ± 0.8 (range 5–9). For the clinical stage, 60% were T1c and 40% T2a. 

The mean prostate weight was 42.9 g ± 18.1 g (range 20 g–100 g). Seven (35%) with prostate weight of 30 g or less, 12 (60%) prostate weight 30.1 g–60 g, and 1 (5%) with prostate weight more than 60 g. 

The mean duration of surgery was 215.3 mins ± 18.7 mins (165 mins–240 mins) with a mean estimated blood loss of 1140.0 mLs ± 534.5 mLs (range 500 mLs–2500 mLs), a median of 1000.0 mLs (Table 2).

The mean perioperative transfusion was 1.7 units ± 1.3 units (range 0.0–4.0 units) with a transfusion rate of 70% (14/20). No blood transfusion in 6 (30%), one unit of blood transfused in 2 (10%), two units in 7 (35%), three units in 3 (15%), and four units in 2 (10%).

The wound drain was removed at a mean of 3.4 days ± 1.9 days (range 1–9 days) with the wound drain removed on postoperative day one in 1 (5%), postoperative day 2 in 2 (10%), and postoperative day 3 in 11 (55%). In 6 (30%) of the patients, the wound drain was removed after three days on the account of persistent drainage.

The pathologic stage, but for two (10%) were higher than the clinical stage. Twelve (60%) of the patients were pT2, 5 (25%) pT3 and 1 (5%) pT4. The two cases were reported as BPH with no malignancy detected after thorough review. The mean pathological Gleason score was 6.8 ± 0.9 (range 6–9) (Table 3).

Five (25%) of the patients had the seminal vesicles infiltrated by the malignant tumour. Three (15%) had positive surgical margins with two involving the prostate apex. For the three patients with positive margins, two of the patients had a course of external beam radiotherapy while one opted for 6 months of hormonal therapy using diethyl stilboestrol with soluble aspirin.

No lymph node involvement was reported. However, in 5 (25%), lymph nodes were not retrieved from the submitted specimen.

In terms of perioperative complications, there was one (5%) rectal injury recognized intraoperatively with successful primary repair and two cases (10%) of wound infection. 

For the functional outcome, seventeen (85%) of the patients had bilateral nerve sparing while 3 (15%) had unilateral nerve sparing. Fourteen (70.0%) of the patients were potent before the radical prostatectomy (IIEF > 16). Eleven (78.6%) of these patients who were potent preoperatively have maintained their potency while 3 (21.4%) have become impotent with no response to sildenafil citrate or tadalafil.

All the patients are continent of urine with most of them being continent by 8 weeks after the procedure. Four (20%) of the patients did not experience any urine incontinence episodes after removal of the urethral catheter. By the end of the first week after removal of their urethral catheter, 11 (55%) were continent of urine, 13 (65%) by the end of the fourth week, 15 (75%) by the end of the eighth week, and 18 (90%) by the end of the twelfth week.

Four (20%) of the patients developed anastomotic stricture that was managed successfully with dilatation/internal urethrotomy; one of these required endoscopic bladder neck resection. Three of these patients that developed anastomotic stricture did not experience any incontinence episodes after removal of their urethral catheters. 

Eighteen (90%) expressed satisfaction with the operation and outcome while 1 (5%) was not satisfied with the outcome at the time of this report. He had bladder neck stenosis that required endoscopic bladder neck resection postoperatively. He is voiding well currently, continent but with erectile dysfunction. 

At a mean followup of 19.5 months, the tPSA of 19 (95%) patients had remained less than 0.4 ng/mL including the patients that had adjuvant radiotherapy and hormonal therapy. One patient with preoperative tPSA of 13.6 ng/mL and pT2a Gleason 4 + 5 at one year followup had the tPSA rise to 45 ng/mL with no evidence of local recurrence or distant metastasis. He has been started on hormonal therapy.

There was no treatment or disease-related death; however, one of the patients died after 18 months of followup (PSA 0.1) due to complications of diabetes mellitus.

## 4. Discussion

Reports indicate differences in the biology of prostate cancer in men of African descent resulting in presentation at an early age, more advanced clinical stage, higher Gleason scores on initial presentation, a poorer five-year survival, and higher recurrence rate [7]. There is also an observed shorter followup period for black Africans diagnosed with prostate cancer [8]. Therefore, access to radical prostatectomy for localized prostate cancer should be of interest including outcomes in those in the West African subregion which offered this potentially curative intervention. With increasing use of PSA screening in some urban communities in West Africa to detect early prostate cancer, strategies to increase the number of surgeons with expertise in this surgery need to be encouraged. This series explores the option of SIU fellowship as a viable option in helping to acquire the skill of radical retropubic prostatectomy with a focus on the early outcomes.

The mean age of the patients in this series of 62.7 yrs which is comparable to the 61 years reported from Jamaica West Indies with 91% of the population being of African ethnicity [9] reflects the choice of the procedure in relatively young and fit patients as contrasts with the general population of black Africans diagnosed with prostate cancer with a mean age of 68.9 yrs in a report from South Africa [8]. As observed in other studies [8–10], most of the patients with prostate cancer in this series presented symptomatically (75%); 40% presenting with lower urinary symptoms, and another 30% with acute retention of urine. Only 25% had their cancers detected through screening due to the absence of effective screening for prostate cancer among the majority of the Ghanaian male population.

The mean preoperative PSA of 16.12 ng/mL was relatively high compared to 10.1 ng/mL reported from the West Indies. But the commonest clinical stage of T1c (60%) noted in this series compares to the report from the West Indies (68%) [9].

The mean operating time of 215.3 mins and estimated blood loss of 1140 mLs compares to reports of established centers, with a mean operative time of 217 and estimated blood loss of 1395 mLs in one study [11]. However, comparing this finding to a contemporary series with mean estimated blood loss of 603 (range 100–3500) [12], the estimated blood loss was higher in this initial series.

The transfusion rate of 70% in this series was rather high compared to a transfusion rate of 4.8% reported by Chang et al. [12]. This could be attributed to the surgical technique as these happen to be the early series in a learning curve as well as limited experience of the anaesthetic team in the procedure of radical retropubic prostatectomy. A defined transfusion trigger can help reduce this rate of transfusion as was observed in a reported retrospective study with transfusion rate being 88.9% in 1988 and dropping to 9.1% in 2002 [13].

Radical prostatectomy offers a better assessment of the true pathological stage tending to be higher than the clinical stage as observed in this series. No malignant lesion was seen in two pathologic specimens though the specimens were subjected to multiple reviews. This calls into question the accuracy of the reports of the initial TRUS prostate core biopsies.

The positive surgical margin of 15% compares to 15.5% reported by Morrison et al. [9] and 11.2 by Eastham et al. [14]. However, seminal vesicle invasion was relatively high by 25% compared to 3.5% in the West Indies study [9]. 

A postoperative complication rate of 15% observed was high compared to 10% in the large series by Catalona et al. [11]. This may reflect the rather limited experience with the procedure as well as the limited number of cases considered in this early series as it had been observed that the incidence of postoperative complications declined significantly with increasing experience of the surgeon [11].

The outcome of the procedure of radical prostatectomy has been judged by the functional results. This includes the assessment of postoperative continence and erectile function [5]. In this study, the erectile function recovery rate in preoperatively potent men was 78.6% compared to 68% of men treated with bilateral nerve sparing surgery in the series by Catalona et al. [11]. All the patients have remained continent of urine. Anastomotic stricture was the commonest complication. Though this was also observed in other studies, 20% incidence was high compared to the 7% reported by Morrison [9]. 

 At a mean followup of 19.5 months, the oncological outcomes assessed by biochemical progression with biochemical recurrence defined as any postoperatively tPSA > 0.4 ng/mL [5] were satisfactory with 95% of the patients having tPSA of less than 0.4 ng/mL and no evidence of local recurrence or metastasis. 

 Considering the patients' initial anxiety over the possible functional results, a satisfaction rate of 90% after the procedure is very encouraging. 

## 5. Conclusion

Open radical retropubic prostatectomy can be safely practiced in West Africa with outcomes comparable to other well-established centers. Lymph node retrieval and anastomotic strictures are the main challenges in this early series. The SIU scholarship offers another avenue for training in radical prostatectomy for sub-Saharan Africa.

## Figures and Tables

**Table 1:** Table showing the patients clinical presentation.

Presentation	No. (%)
Lower urinary obstructive symptoms	8 (40)
Acute retention of urine	6 (30)
Routine screening by PSA	5 (25)
Pelvic pain	1 (5)

Total	20 (100)

**Table 2:** Estimated blood loss during radical prostatectomy.

Estimated blood loss (mls)	No. (%)
<500	0 (0.0)
500–999	8 (40.0)
1000–1499	7 (35.0)
1500–1999	3 (15.0)
2000–2499	0 (0.0)
≥2500	2 (10.0)

**Table 3:** Table showing clinical stage at diagnosis (trus biopsy) versus pathological stage of the prostate cancer.

Serial number	Age (yrs)	tPSA (ng/mL)	Clinical stage	Pathological stage
1	63	7.60	T1c 3 + 3	BPH
2	72	7.90	T2a 3 + 4	T3b 4 + 3 (no lymph nodes)
3	55	62.20	T1c 3 + 3	T3b 4 + 3 N0
4	69	11.10	T1c 3 + 4	T3b 4 + 5 N0
5	61	8.30	T2a 3 + 2	BPH (no lymph nodes)
6	62	2.45	T2a 3 + 3	T2b 3 + 3 N0
7	63	28.00	T1c 4 + 3	T4 3 + 3 N0
8	63	7.60	T1c 3 + 3	T2a 3 + 3 N0
9	55	13.60	T1c 4 + 3	T2a 4 + 5 (no lymph nodes)
10	66	8.80	T2a 3 + 3	T3b 4 + 3 N0
11	58	39.39	T2a 3 + 3	T2b 4 + 3 N0
12	60	7.01	T1c 3 + 3	T2a 3 + 3 N0
13	60	12.00	T2a 3 + 3	T2b 3 + 3 N0
14	72	13.00	T2a 4 + 3	T2b 4 + 3 N0
15	51	14.03	T1c 4 + 3	T2a 4 + 3 N0
16	59	9.00	T1c 4 + 3	T2c 3 + 4 (no lymph nodes)
17	67	13.99	T1c 4 + 5	T2c 3 + 3 (no lymph nodes)
18	65	19.00	T1c 3 + 3	T2b 3 + 3 N0
19	64	21.17	T2a 4 + 3	T3a 3 + 4 N0
20	69	16.30	T1c 3 + 4	T2a 3 + 4 N0
